# Effect of the CALHM1 G330D and R154H Human Variants on the Control of Cytosolic Ca^2+^ and Aβ Levels

**DOI:** 10.1371/journal.pone.0112484

**Published:** 2014-11-11

**Authors:** Valérie Vingtdeux, Jessica E. Tanis, Pallavi Chandakkar, Haitian Zhao, Ute Dreses-Werringloer, Fabien Campagne, J. Kevin Foskett, Philippe Marambaud

**Affiliations:** 1 Litwin-Zucker Research Center for the Study of Alzheimer's Disease, The Feinstein Institute for Medical Research, Manhasset, NY, United States of America; 2 Department of Physiology, Perelman School of Medicine, University of Pennsylvania, Philadelphia, PA, United States of America; 3 The HRH Prince Alwaleed Bin Talal Bin Abdulaziz Alsaud Institute for Computational Biomedicine, The Weill Cornell Medical College, New York, NY, United States of America; 4 Department of Cell and Developmental Biology, Perelman School of Medicine, University of Pennsylvania, Philadelphia, PA, United States of America; University of S. Florida College of Medicine, United States of America

## Abstract

CALHM1 is a plasma membrane voltage-gated Ca^2+^-permeable ion channel that controls amyloid-β (Aβ) metabolism and is potentially involved in the onset of Alzheimer's disease (AD). Recently, Rubio-Moscardo et al. (*PLoS One* (2013) 8: e74203) reported the identification of two CALHM1 variants, G330D and R154H, in early-onset AD (EOAD) patients. The authors provided evidence that these two human variants were rare and resulted in a complete loss of CALHM1 function. Recent publicly available large-scale exome sequencing data confirmed that R154H is a rare CALHM1 variant (minor allele frequency (MAF)  = 0.015%), but that G330D is not (MAF  = 3.5% in an African American cohort). Here, we show that both CALHM1 variants exhibited gating and permeation properties indistinguishable from wild-type CALHM1 when expressed in *Xenopus* oocytes. While there was also no effect of the G330D mutation on Ca^2+^ uptake by CALHM1 in transfected mammalian cells, the R154H mutation was associated with defects in the control by CALHM1 of both Ca^2+^ uptake and Aβ levels in this cell system. Together, our data show that the frequent CALHM1 G330D variant has no obvious functional consequences and is therefore unlikely to contribute to EOAD. Our data also demonstrate that the rare R154H variant interferes with CALHM1 control of cytosolic Ca^2+^ and Aβ accumulation. While these results strengthen the notion that CALHM1 influences Aβ metabolism, further investigation will be required to determine whether CALHM1 R154H, or other natural variants in CALHM1, is/are associated with EOAD.

## Introduction

Alzheimer's disease (AD) is a progressive neurodegenerative disorder leading to the most common form of dementia in elderly people. Histological studies of the AD brain have revealed pathological changes triggered by two classical lesions, the senile plaques and neurofibrillary tangles [1], [2]. Senile plaques result from the accumulation of amyloid-β (Aβ), a series of peptides produced by sequential endoproteolysis of the amyloid precursor protein (APP) by β- and γ-secretases [3], [4]. APP is genetically linked to early-onset familial forms of AD and Aβ is considered to be a causative factor in AD [5]. The etiology of AD is influenced by a strong genetic heterogeneity. Rare autosomal dominant mutations cause early-onset familial AD, whereas complex interactions between different genetic variants and environmental factors modulate the risk for the vast majority of late-onset AD cases [6]–[8].

*Calcium homeostasis modulator 1* (*CALHM1*) [9] was identified by a tissue-specific gene expression profiling approach [10], [11] that screened for genes located on susceptibility loci for AD and that are preferentially expressed in the hippocampus, a brain region affected early in AD [12]. CALHM1 is a plasma membrane Ca^2+^-permeable ion channel regulated by voltage and extracellular Ca^2+^ (Ca^2+^_o_) levels [9], [13]–[15]. The exact function of CALHM1 in the brain is not completely understood, but recent evidence has revealed that CALHM1 controls neuronal intracellular Ca^2+^ (Ca^2+^_i_) homeostasis and signaling, as well as Ca^2+^-dependent neuronal excitability [13], [14].

We initially proposed that the naturally occurring P86L variant in *CALHM1* (rs2986017) was associated in European cohorts with both AD risk and an earlier age-at-onset of AD [9]. Reports of both refutation and confirmation of the association with AD risk in independent genetic studies followed the original results [16]. A meta-analysis of all published studies has now shown that *CALHM1 per se* has no significant impact on AD risk and is thus likely not a robust independent risk gene for AD [16]. However, the meta-analysis confirmed the association of *CALHM1* with AD age-at-onset [16]. In support of the idea that CALHM1 might be involved in the pathological process of AD, we have reported that CALHM1 activation triggers a Ca^2+^-dependent pathway that suppresses extracellular Aβ accumulation in cell lines [9]. Moreover, two independent genetic studies have showed that the CALHM1 P86L variant influences Aβ levels in human cerebrospinal fluid [17], [18], but see also [19]. *In vitro* functional studies further demonstrated that the P86L variant – through a mechanism yet to be determined [14] – caused a partial loss of function by inhibiting the effect of CALHM1 on Ca^2+^ influx and Aβ repression [9], [13], [14], [20], [21]. Altogether these results support the notion that CALHM1 controls Aβ metabolism and AD pathogenesis.

In a recent study [20], Rubio-Moscardo et al. reported the identification of two natural variants in CALHM1 that occurred in early-onset AD (EOAD) patients. The two CALHM1 variants, G330D and R154H, were identified by sequencing *CALHM1* coding regions in three independent series comprising a total of 284 EOAD patients and 326 controls. The authors found that the G330D and R154H variants were associated with a complete loss of CALHM1 control of Ca^2+^ influx in cell lines, suggesting that these variations interfere with CALHM1 function and may thus contribute to the risk of EOAD [20]. In the current study, we have reassessed the functionality of the CALHM1 G330D and R154H variants on channel gating and Ca^2+^ permeability by using electrophysiological recordings and Ca^2+^_i_ measurements in CALHM1-expressing oocytes and mammalian cells, respectively. While there was no effect of the G330D mutation on CALHM1 function in any of our experimental approaches, we found that the R154H mutation resulted in a partial inhibition of CALHM1-dependent Ca^2+^ uptake in cell lines.

In the context of characterizing CALHM1 function, Rubio-Moscardo et al. failed to observe an inhibition of Aβ accumulation by CALHM1 expression using a modified version of the originally described protocol of CALHM1 activation (hereafter referred to as the “Ca^2+^ add-back” protocol [9]), which consists of transiently lowering Ca^2+^_o_ and then adding it back at physiological concentration [13], [14], [21], [22]. Here, we have also reassessed the effect of CALHM1 activation on Aβ levels in cell lines by testing different Ca^2+^ add-back protocols, including the modified version used by Rubio-Moscardo et al. In all conditions, we confirmed the robust and sustained repressing effect of CALHM1 activation on Aβ accumulation. In line with the effect of the two CALHM1 variants on CALHM1-mediated Ca^2+^ influx, we further demonstrate that the G330D variant has no functional effect on the control of Aβ levels by CALHM1, whereas the R154H variant is associated with a partial loss of CALHM1 function.

## Results and Discussion

### CALHM1 variant frequency in a large-scale exome sequencing database

We interrogated the NHLBI Exome Sequencing Project (ESP) database to determine the frequency of the CALHM1 R154H and G330D variants (see Methods). The R154H variant (10∶105218048, rs371900329) was found in a single individual across the entire ESP cohort (minor allele frequency (MAF)  = 0.015%). In contrast, the G330D variation (10∶105215071, rs114015468) was found in one individual of European-American ethnicity and in 151/4,255 individuals of African-American ethnicity (a 3.55% MAF in the latter population). Therefore, sequencing analysis of these large human cohorts show that CALHM1 R154H is a rare variation, but that CALHM1 G330D, with a MAF above 1%, is not.

### The R154H and G330D variants do not alter CALHM1 gating or Ca^2+^ permeability

**Figure 1:**
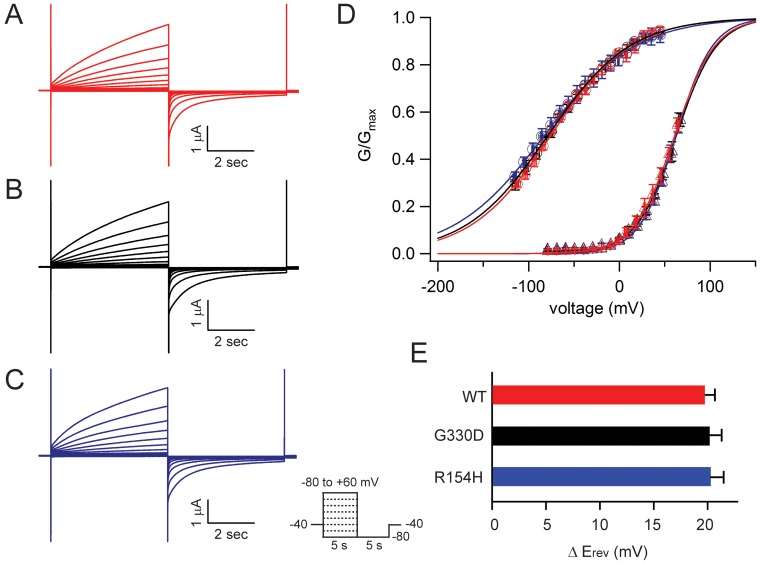
Effect of the R154H and G330D variants on CALHM1 gating and Ca^2+^ permeability. **A–C**. Currents observed in oocytes expressing (**A**) WT-CALHM1, (**B**) G330D-CALHM1, and (**C**) R154H-CALHM1 in standard bath solution containing 2 mM Ca^2+^_o_ in response to voltage pulses from −80 mV to +60 mV; holding potential −40 mV. **D**. Following a series of voltage pulses, currents at −80 mV were measured to determine G-V relations in the presence and absence of Ca^2+^_o_. For each oocyte, G_max_ was determined by fitting 0 mM Ca^2+^_o_ data with a Boltzmann function; all currents were then normalized to G_max_. Normalized data were fit with Boltzmann functions with the assumption that Ca^2+^ does not affect G_max_ [14], [15]. WT-CALHM1 0 mM Ca^2+^_o_ (red circles) V_0.5_ =  −75.9 mV; R154H-CALHM1 0 mM Ca^2+^_o_ (blue circles) V_0.5_ =  −82.8 mV; G330D-CALHM1 0 mM Ca^2+^_o_ (black circles) V_0.5_ =  −78.8 mV; WT-CALHM1 2 mM Ca^2+^_o_ (red triangles) V_0.5_ = 60.8 mV; R154H-CALHM1 2 mM Ca^2+^_o_ (blue triangles) V_0.5_ = 62.4 mV; G330D-CALHM1 2 mM Ca^2+^_o_ (black triangles) V_0.5_ = 62.8 mV. **E**. Changes in E_rev_ resulting from changing from 0 to 2 mM Ca^2+^_o_ solution in oocytes expressing WT-CALHM1 (red), G330D-CALHM1 (black), and R154H-CALHM1 (blue). n = 4–6 oocytes for each condition; Error bars, SE.

Heterologously expressed CALHM1 exhibits properties consistent with it being a Ca^2+^-permeable ion channel regulated by voltage and Ca^2+^_o_ [14], [15]. Rubio-Moscardo et al. observed that the R154H and G330D variants resulted in a complete loss of CALHM1-dependent Ca^2+^ influx in transfected mammalian cells [20]. To determine if R154H or G330D alters CALHM1 gating or Ca^2+^ permeability, we recorded whole cell currents from *Xenopus* oocytes injected with wild-type (WT)-CALHM1, R154H-CALHM1, and G330D-CALHM1 cRNA. In the presence of 2 mM Ca^2+^_o_, membrane depolarization of oocytes expressing WT-CALHM1 or the CALHM1 variants resulted in slowly-activating outward currents that deactivated upon stepping to hyperpolarized voltages (Figs. 1A–C). Using the same conditions, similar currents were not observed in water-injected oocytes (data not shown, see also Ref. [14]). This indicates that R154H-CALHM1 and G330-CALHM1 form functional voltage-dependent channels.

Lowering Ca^2+^_o_ resulted in a left shift in the apparent conductance-voltage (G-V) relationship for CALHM1, allowing channels to open at resting membrane potentials (Fig. 1D, see also Ref. [14]). To determine if R154H-CALHM1 and G330-CALHM1 are also regulated by Ca^2+^_o_, we recorded currents from CALHM1-expressing oocytes in divalent-free and 2 mM Ca^2+^_o_ containing solutions, and analyzed the apparent G-V relationships. For WT-CALHM1, 2 mM Ca^2+^_o_ right shifted the apparent G-V relationship by 136.7 mV from a half-activation voltage (V_0.5_) of –75.9 mV in 0 mM Ca^2+^_o_ to +60.8 mV in 2 mM Ca^2+^_o_ (Fig. 1D). Similarly, the presence of 2 mM Ca^2+^_o_ caused 145.2 mV and 141.6 mV right shifts in the apparent G-V relationships for R154H-CALHM1 and G330D-CALHM1, respectively (Fig. 1D). These results suggest that R154H-CALHM1 and G330D-CALHM1 are regulated by both voltage and Ca^2+^_o_, with gating properties indistinguishable from WT-CALHM1.

We then asked whether the lack of Ca^2+^ influx observed by Rubio-Moscardo et al. in cell lines expressing R154H-CALHM1 and G330D-CALHM1 was due to alterations in Ca^2+^ permeability. To assess if R154H-CALHM1 and G330D-CALHM1 are permeable to Ca^2+^, we measured reversal potentials (E_rev_) in solutions containing 0 or 2 mM Ca^2+^_o_ with constant NaCl. The presence of Ca^2+^_o_ resulted in a shift in E_rev_ to more depolarized voltages for WT-CALHM1, R154H-CALHM1, and G330D-CALHM1 (Fig. 1E). This shift in E_rev_ was not significantly different for the two variants compared to WT-CALHM1, suggesting that relative Ca^2+^ permeability was not altered. In conclusion, we were unable to detect any difference in the gating or Ca^2+^ permeability for the R154H and G330D variants, compared to WT-CALHM1.

### Effect of the CALHM1 G330D and R154H variants on Ca^2+^ influx in mammalian cells

**Figure 2:**
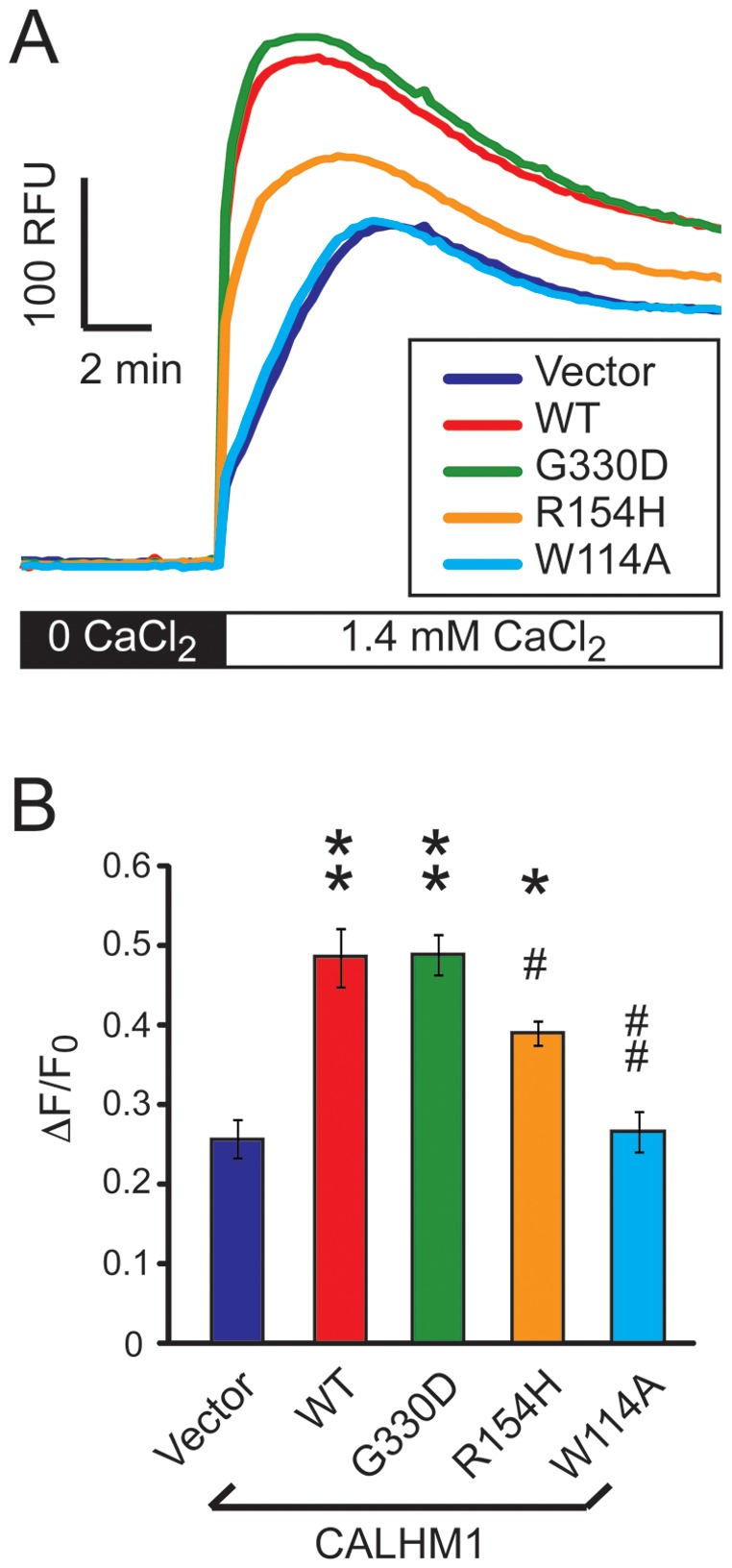
Effect of the CALHM1 G330D and R154H variants on Ca^2+^ influx in mammalian cells. **A**. Ca^2+^_i_ measurements with Fluo-4 loading and Ca^2+^ add-back in HT-22 cells transiently transfected with WT-, G330D-, R154H-, and W114A-CALHM1 or empty vector. Cells were incubated in Ca^2+^-free buffer (0 CaCl_2_) for 30 min, and then challenged with physiological Ca^2+^_o_ concentration (1.4 mM CaCl_2_) to monitor the restoration of Ca^2+^_i_ levels. RFU, relative fluorescence units. **B**. Peak of Ca^2+^_i_ concentration measurements after Ca^2+^ add-back expressed as ΔF/F_0_. Error bars, SEM. *P<0.01, **P<0.001, relative to vector-transfected cells; ^#^ *P*<0.01, ^##^ *P*<0.001, relative to WT-CALHM1-transfected cells (ANOVA with Bonferroni correction, n = 3 independent experiments as in (**A**)).

To further investigate the effect of the G330D and R154H mutations on CALHM1 function, we evaluated CALHM1-mediated Ca^2+^ influx by measuring Ca^2+^_i_ levels in transfected hippocampal HT-22 cells loaded with the fluorescent Ca^2+^ indicator Fluo-4. Removal and subsequent add-back of Ca^2+^_o_ results in a CALHM1-mediated increase in Ca^2+^_i_ levels [9]. Upon Ca^2+^ add-back stimulation, we found that G330D-CALHM1 produced the same elevation in Ca^2+^_i_ levels as WT-CALHM1 (Figs. 2A and 2B). In contrast, R154H significantly inhibited CALHM1-mediated Ca^2+^ influx in these conditions (Figs. 2A and 2B). R154H-CALHM1 only exhibited a partial loss of function compared to the dead mutant W114A-CALHM1 that, as expected [13], fully inhibited CALHM1-mediated Ca^2+^ influx (Figs. 2A and 2B). These results show that the G330D mutation did not alter CALHM1 function, while the R154H mutation resulted in a partial loss of function in mammalian cells.

### Reevaluation of the effect of CALHM1 activation on Aβ levels

In our original study [9], we showed that Ca^2+^ add-back in two CALHM1 expressing neuroblastoma cell lines had an inhibitory effect on extracellular Aβ accumulation. Expression of CALHM1 in N2a cells decreased Aβ levels, while silencing of CALHM1 in SH-SY5Y cells increased Aβ levels, upon Ca^2+^ add-back [9]. The decrease in Aβ levels observed in CALHM1-transfected cells was accompanied by enhanced secretion of sAPPα [9], the product of the non-amyloidogenic processing of APP [23]. In the context of characterizing the functionality of the identified CALHM1 variants, Rubio-Moscardo et al. failed to observe any effect of CALHM1 expression on extracellular Aβ accumulation [20]. The authors tested CALHM1-transfected HEK293 cells and used a modified version of the original Ca^2+^ add-back protocol, which extended the Ca^2+^ omission step to 30 min [20], compared to 1–10 min in the previous studies [9], [13], [14], [21], [22]. A more significant difference in Rubio-Moscardo et al. protocol relates to the Aβ secretion time period after Ca^2+^ add-back: 6 hr [20] vs. 60 min in our protocol [9]. In addition, Rubio-Moscardo et al. performed the Ca^2+^ add-back protocol in the presence of 1% FBS, an ingredient that was omitted in our previous study.

**Figure 3:**
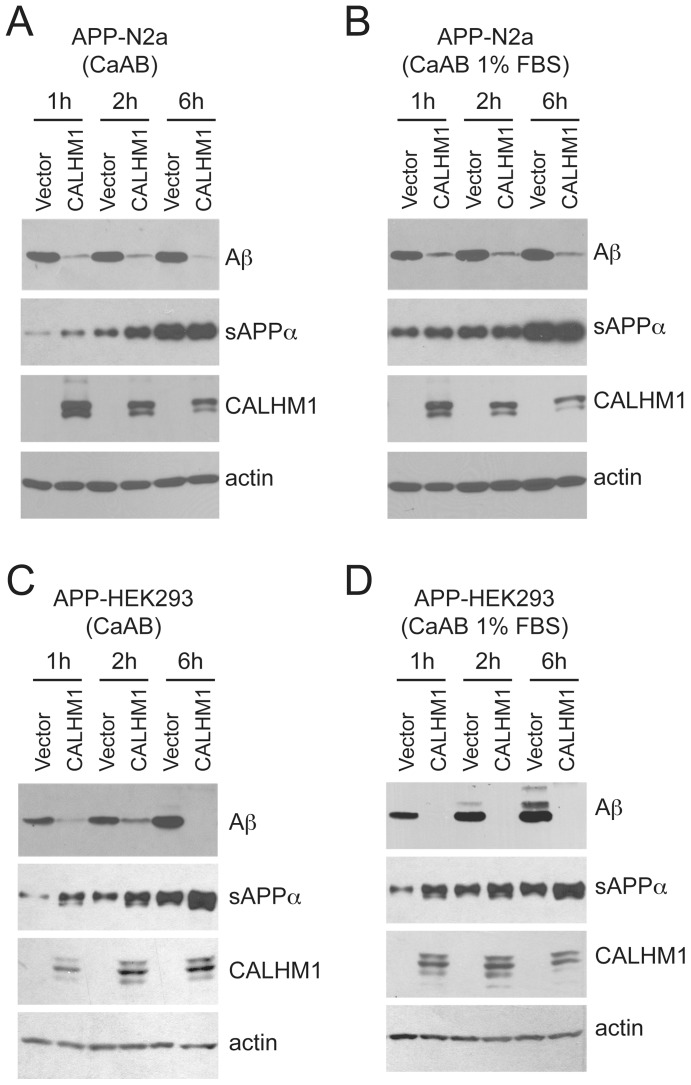
Effect of CALHM1 activation on Aβ levels. **A–D**. APP-N2a (**A** and **B**) and APP-HEK293 (**C** and **D**) cells transfected with empty vector or WT-CALHM1 were subjected to Ca^2+^ add-back conditions in the absence (CaAB, **A** and **C**) or presence of 1% FBS (CaAB 1% FBS, **B** and **D**). Extracellular Aβ and sAPPα were analyzed by WB after the indicated periods of secretion. Cell lysates were probed using anti-Myc and anti-actin antibodies to detect CALHM1 and actin, respectively.

In this context, we reassessed the effect of CALHM1 expression on Aβ levels not only in N2a cells, but also in HEK293 cells, and by using Ca^2+^ add-back conditions that included: (i) a 30 min Ca^2+^ omission, (ii) 1% FBS, and (iii) Ca^2+^ add-back secretion time periods that extended to 6 hr. In all tested conditions, Ca^2+^ add-back in CALHM1 expressing cells consistently inhibited the accumulation of extracellular Aβ (Fig. 3). Specifically, the amount of time allowed for secretion following Ca^2+^ add-back (Figs. 3A and 3C) and the presence of 1% FBS (Figs. 3B and 3D) did not affect CALHM1-mediated inhibition of Aβ accumulation. In addition, the previously reported CALHM1-mediated stimulation of sAPPα release [9] was observed in nearly all tested conditions (Fig. 3), with the exception of the N2a cells incubated without FBS (for the 6 hr secretion time point, see Fig. 3A) and at all time points with FBS present (Fig. 3B). These data show that, in different cell lines, CALHM1 expression and its activation by the Ca^2+^ add-back condition generate a potent and long-lasting repressing effect on extracellular Aβ accumulation. The differences in the Ca^2+^ add-back protocol used by Rubio-Moscardo et al. can therefore not explain the disparity in the results observed their study [20]. We note that all transfections performed by Rubio-Moscardo et al. did not include assessment of protein expression efficiency after transfection, raising concerns about the functional expression of CALHM1 in their system. Indeed, Ca^2+^ measurements are very sensitive and could have detected low CALHM1 expression (e.g., in the presence of poor transfection efficiency) that would fail to translate into an effect on Aβ levels.

### Effect of the CALHM1 G330D and R154H variants on Aβ levels

**Figure 4:**
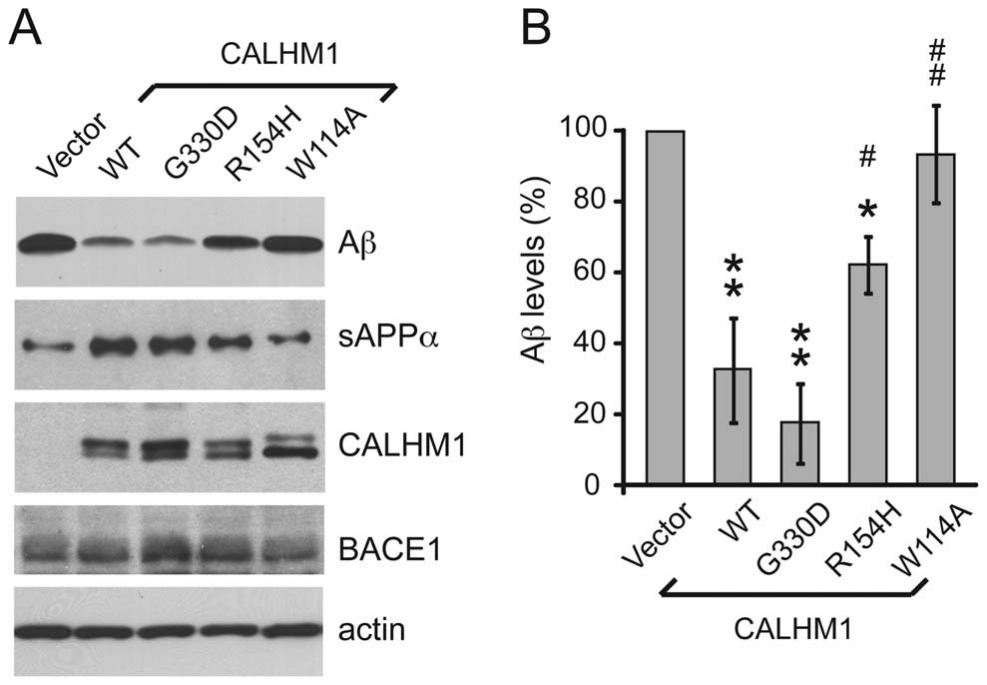
Effect of the CALHM1 G330D and R154H variants on Aβ levels. **A**. APP-N2a cells were transiently transfected with empty vector or WT-, G330D-, and R154H-CALHM1, as well as W114A-CALHM1 (CALHM1 dead mutant control, see Ref. [13]). Cells were then subjected to Ca^2+^ add-back as in Fig. 3A. Extracellular Aβ and sAPPα were analyzed by WB after 1 hr of secretion. Cell lysates were probed using anti-Myc, anti-BACE1, and anti-actin antibodies to detect CALHM1, BACE1, and actin, respectively. **B**. Densitometric analyses and quantification of Aβ levels in 3 independent measurements as in (**A**). Error bars, SEM; *P<0.01, **P<0.001, relative to vector-transfected cells; ^#^ *P*<0.05, ^##^ *P*<0.001, relative to WT-CALHM1-transfected cells (ANOVA with Bonferroni correction).

To determine whether the R154H and G330D variants affect Aβ accumulation, we exposed transfected N2a cells to the 1 hr Ca^2+^ add-back protocol, as in Fig. 3A. We observed that G330D-CALHM1 produced the same inhibitory effect on Aβ accumulation as WT-CALHM1 (Figs. 4A and 4B). In contrast, expression of R154H-CALHM1 was less effective in reducing Aβ levels, compared to WT-CALHM1 (Figs. 4A and 4B). In addition, WT-CALHM1 and G330D-CALHM1 produced comparable stimulation of sAPPα secretion, while there was less sAPPα secreted from cells expressing R154H-CALHM1 (Fig. 4A). These effects on APP metabolism did not result from significant changes in the expression levels of the different CALHM1 mutants (Fig. 4A). These data, which are in line with the effect of the two CALHM1 variants on CALHM1-mediated Ca^2+^ influx in mammalian cells (Fig. 2), further demonstrate that the G330D variant has no obvious functional effect on CALHM1, whereas the R154H variant is associated with a partial loss of CALHM1 function.

Previous work has shown that Ca^2+^_i_ disturbances can affect APP processing and Aβ production [24], [25]. Notably, expression of BACE1 β-secretase is controlled by different Ca^2+^-dependent transcriptional pathways [26], [27]. To determine whether CALHM1-mediated Ca^2+^ influx influences BACE1 expression, we measured BACE1 levels by western blotting (WB) in cells transfected with WT or mutated CALHM1. No significant differences in BACE1 levels were observed in these conditions (Fig. 4A), showing that CALHM1 did not affect Ca^2+^-dependent BACE1 expression. Therefore, CALHM1 influenced Aβ levels by a mechanism independent of BACE1 transcriptional control.

Together our data show that, while R154H-CALHM1 exhibits normal channel properties at the plasma membrane (Fig. 1), it impairs the overall control by CALHM1 of Ca^2+^ cell uptake (Fig. 2) and APP metabolism (Figs. 3 and 4). These results suggest that R154H does not prevent the formation of a fully functional channel, but instead may act by interfering with CALHM1 trafficking and/or its maturation to reduce the expression levels or stability of CALHM1 at the plasma membrane. The effect of R154H on CALHM1-mediated Ca^2+^ influx could also indirectly be influenced by other plasma membrane Ca^2+^ channels, such the store-operated Ca^2+^ (SOC) channels. Our previous work has failed, however, to observe an effect of CALHM1 activation on SOC entry [14] and SOC channel inhibition did not interfere with CALHM1-mediated Ca^2+^ influx [9]. Selective blockage of other common plasma membrane Ca^2+^ channels in excitable cells, such as the voltage-gated Ca^2+^ channels (VGCCs), was also without effect on CALHM1-mediated Ca^2+^ influx [9]. Collectively, these data suggest that CALHM1 does not influence SOC channels or VGCCs. The effect of the CALHM1 R154H mutation is thus unlikely to be due to defects in the response of these other channels. Nevertheless, we cannot exclude the possibility at this point that other unidentified pathways of Ca^2+^ re-entry might be involved in this mechanism. The effect of the R154H mutation on CALHM1 is therefore likely to be complex and further investigation will be required to determine the precise mechanisms whereby this mutation interferes with CALHM1 function.

## Conclusions

In the present work, we used electrophysiology and cell biology approaches to show that: (i) CALHM1 G330D is a frequent variant with no functional consequences on CALHM1 channel properties and is therefore unlikely to contribute to the risk of EOAD; and (ii) CALHM1 R154H is a rare variant that causes a partial loss of CALHM1 function and increases Aβ levels in cell lines. These results motivate further investigation aimed at determining whether CALHM1 R154H, or other functional natural variants in CALHM1, is/are associated with EOAD.

## Materials and Methods

### Antibodies and plasmids

Anti-myc antibody (71D01) was from Cell Signaling Technology, anti-actin antibody from BD Transduction Laboratories, anti-Aβ_1–17_ (6E10) antibody from Covance, and anti-BACE1 antibody from Abcam. Myc-tagged WT- and W114A-CALHM1 in pcDNA3.1 vectors, as well as WT-CALHM1 in the pBF oocyte expression vector, were described previously [9], [13]. G330D- and R154H-CALHM1 were created using Quickchange II site-directed mutagenesis kit (Agilent Technologies) and confirmed by sequencing.

### Cell lines and transfections

The cell lines used in this study were described previously [13], [23]. HT-22 cells were kindly provided by Dr. D. Schubert (Salk Institute, La Jolla, CA). HEK293 cells stably transfected with human APP_695_ (APP-HEK293) were provided by Dr. Luciano D'Adamio (Albert Einstein College of Medicine, Bronx, NY). N2a cells stably transfected with human APP_695_ (APP-N2a) were obtained from Dr. Gopal Thinakaran (university of Chicago, Chicago, IL). HT-22 and APP-HEK293 cells were maintained in Dulbecco's modified Eagle's medium (DMEM) supplemented with 10% fetal bovine serum (FBS, HyClone, Thermo Fisher Scientific Inc.), 2 mM L-glutamine, and penicillin/streptomycin (Life Technologies) at 37°C under 5% CO_2_. APP-N2a cells were maintained in DMEM/Opti-MEM (1∶1) supplemented with 10% FBS, 2 mM L-glutamine, and penicillin/streptomycin at 37°C under 5% CO_2_. Cells were transiently transfected with CALHM1 cDNAs using Lipofectamine 2000, as per the manufacturer's instructions (Life Technologies). Transfections were performed in complete culture medium for APP-N2a and APP-HEK293 cells, or in Opti-MEM for HT-22 cells. For HT-22 cells, Opti-MEM was replaced 5 hr post-transfection with complete culture medium. All cell lines were processed 24 hr after transfection.

### Ca^2+^ add-back assay

Ca^2+^ add-back was performed as described previously [9], [13], with the following modifications: Cells were incubated for 30 min in Ca^2+^/Mg^2+^-free Hank's balanced salt solution (HBSS) supplemented with 20 mM HEPES buffer, 0.5 mM MgCl_2_, and 0.4 mM MgSO_4_. Ca^2+^ was then added back for the indicated periods of time to a final concentration of 1.4 mM in the absence or presence of 1% FBS.

### Intracellular Ca^2+^ measurements

Ca^2+^_i_ levels were measured using Fluo-4 (Fluo-4 NW Ca^2+^ Assay kit, Life Technologies) as described previously [9], [13]. Briefly, HT-22 cells were transfected with WT-CALHM1, CALHM1 mutants, or empty vector, as described above. Twenty-four hours post-transfection, cells were loaded with Fluo-4 and fluorescence measurements were performed at room temperature with a Tecan Genius Pro plate reader at 480 nm excitation and 535 nm emission. After fluorescence measurements, cells were washed and analyzed by WB to analyze CALHM1 protein levels.

### WB analyses

Cell extracts (5–20 µg, depending on the primary antibody used) were analyzed by SDS-PAGE using the antibodies listed above. Secreted total Aβ and sAPPα levels were analyzed by WB, as described previously [28], [29]. Briefly, 20 µL of medium was electrophoresed on 16.5% Tris-tricine gels (for Aβ WB) or 10% Tris-HCl gels (sAPPα WB) and then transferred onto nitrocellulose membranes. For Aβ WB, membranes were microwaved for 5 min in PBS. Membranes were then blocked in 5% fat-free milk in TBS and incubated overnight at 4°C with 6E10 antibody (1∶1000 in SuperBlock Blocking Buffer, Thermo Fisher Scientific). A standard ECL detection procedure was then used.

### Electrophysiology in *Xenopus* oocytes

All procedures involving the use of *Xenopus laevis* were approved by the University of Pennsylvania Institutional Animal Care and Use Committee. Adult female *Xenopus laevis* obtained from Xenopus One were maintained in existing university facilities. To obtain oocytes, *Xenopus laevis* were first anaesthetized by immersion in 0.15% tricaine for 15 minutes. Once anaesthetized, frogs were placed supine, a 1.0 cm incision was made in the lower abdominal quadrant through which part of an ovary was raised and removed and then the incision was closed with 4–0 Reli sutures (MYCO Medical). Oocytes were defolliculated with type 2 collagenase (Worthington Biochemical). No *Xenopus laevis* frogs or embryos were sacrificed for this study.

CALHM1 cRNAs were synthesized from MluI-linearized plasmids with SP6 RNA polymerase (mMessage mMachine kit, Ambion). CALHM1 cRNA (2 ng) was injected into stage IV–VI *Xenopus laevis* oocytes with *Xenopus* connexin-38 antisense oligonucleotide (ASO; 80 ng). Injected oocytes were incubated for 1 day at 16°C in ND96 medium (96 mM NaCl, 2 mM KCl, 1.8 mM CaCl_2_, 1 mM MgCl_2_, 2.5 mM Na-pyruvate, 1X penicillin-streptomycin, pH 7.6) and then injected with 50 nl of a 20 mM BAPTA, 10 mM Ca^2+^ solution at least 30 min before recording to prevent activation of endogenous Ca^2+^-activated Cl^−^ currents. Data were acquired at 1 kHz using an OC-725C amplifier (Warner Instrument Corp.) with a 16-bit A/D converter (Instrutech ITC-16). Electrodes were made with TW100F-6 glass (World Precision Instruments, Inc.) using a model P-87 Sutter Instrument Co. micropipette puller and then filled with 3 M KCl.

Standard bath solutions (pH 7.2) contained (in mM) 100 Na^+^, 100 Cl^−^, 2 K^+^, and 10 HEPES with 2 Ca^2+^ or 0.5 EGTA and 0.5 EDTA to create the divalent cation-free solution. To determine conductance-voltage relations in the presence and absence of Ca^2+^_o_, currents at −80 mV were measured following a series of voltage pulses, as described before [14], [15]. For the permeability experiments, standard bath solution (pH 7.2) contained (in mM) 17 Na^+^, 10 Cl^−^ ±2 Ca^2+^. Instantaneous I–V recordings were analyzed to determine the reversal potential, as described before [14], [15]. Recordings were analyzed with Igor Pro.

### Variation allele frequency analysis

We interrogated the NHLBI Exome Sequencing Project (ESP) release ESP6500SI-V2 using the Exome Variant Server [30]. The ESP measured genotypes in a population of 6,503 individuals across several cohorts using an exome-sequencing assay. Exome sequencing makes it possible to observe genomic sequence over the covered exonic regions and to call rare variants in these regions.

## References

[1] Serrano-Pozo A, Frosch MP, Masliah E, Hyman BT (2011) Neuropathological alterations in Alzheimer disease. Cold Spring Harb Perspect Med 1: a006189. 22229116 10.1101/cshperspect.a006189 PMC3234452

[2] Duyckaerts C, Delatour B, Potier MC (2009) Classification and basic pathology of Alzheimer disease. Acta Neuropathol 118: 5–36. 19381658 10.1007/s00401-009-0532-1

[3] De Strooper B, Vassar R, Golde T (2010) The secretases: enzymes with therapeutic potential in Alzheimer disease. Nat Rev Neurol 6: 99–107. 20139999 10.1038/nrneurol.2009.218 PMC2879045

[4] Marambaud P, Robakis NK (2005) Genetic and molecular aspects of Alzheimer's disease shed light on new mechanisms of transcriptional regulation. Genes Brain Behav 4: 134–146. 15810902 10.1111/j.1601-183X.2005.00086.x

[5] Selkoe D, Mandelkow E, Holtzman D (2012) Deciphering Alzheimer disease. Cold Spring Harb Perspect Med 2: a011460. 22315723 10.1101/cshperspect.a011460 PMC3253026

[6] Goate A, Hardy J (2012) Twenty years of Alzheimer's disease-causing mutations. J Neurochem 120 Suppl 1 3–8. 22122678 10.1111/j.1471-4159.2011.07575.x

[7] Lambert JC, Amouyel P (2007) Genetic heterogeneity of Alzheimer's disease: complexity and advances. Psychoneuroendocrinology 32 Suppl 1 S62–70. 17659844 10.1016/j.psyneuen.2007.05.015

[8] Ertekin-Taner N (2010) Genetics of Alzheimer disease in the pre- and post-GWAS era. Alzheimers Res Ther 2: 3. 20236449 10.1186/alzrt26 PMC2874262

[9] Dreses-Werringloer U, Lambert JC, Vingtdeux V, Zhao H, Vais H, et al (2008) A polymorphism in CALHM1 influences Ca2+ homeostasis, Abeta levels, and Alzheimer's disease risk. Cell 133: 1149–1161. 18585350 10.1016/j.cell.2008.05.048 PMC2577842

[10] Aguilar D, Skrabanek L, Gross SS, Oliva B, Campagne F (2008) Beyond tissueInfo: functional prediction using tissue expression profile similarity searches. Nucleic Acids Res 36: 3728–3737. 18483083 10.1093/nar/gkn233 PMC2441795

[11] Skrabanek L, Campagne F (2001) TissueInfo: high-throughput identification of tissue expression profiles and specificity. Nucleic Acids Res 29: E102–102. 11691939 10.1093/nar/29.21.e102 PMC60201

[12] de Leon MJ, Mosconi L, Blennow K, DeSanti S, Zinkowski R, et al (2007) Imaging and CSF studies in the preclinical diagnosis of Alzheimer's disease. Ann N Y Acad Sci 1097: 114–145. 17413016 10.1196/annals.1379.012

[13] Dreses-Werringloer U, Vingtdeux V, Zhao H, Chandakkar P, Davies P, et al (2013) CALHM1 controls the Ca^2^ ^+^-dependent MEK, ERK, RSK and MSK signaling cascade in neurons. J Cell Sci 126: 1199–1206. 23345406 10.1242/jcs.117135 PMC4481642

[14] Ma Z, Siebert AP, Cheung KH, Lee RJ, Johnson B, et al (2012) Calcium homeostasis modulator 1 (CALHM1) is the pore-forming subunit of an ion channel that mediates extracellular Ca2+ regulation of neuronal excitability. Proc Natl Acad Sci U S A 109: E1963–1971. 22711817 10.1073/pnas.1204023109 PMC3396471

[15] Tanis JE, Ma Z, Krajacic P, He L, Foskett JK, et al (2013) CLHM-1 is a functionally conserved and conditionally toxic Ca2+-permeable ion channel in Caenorhabditis elegans. J Neurosci 33: 12275–12286. 23884934 10.1523/JNEUROSCI.5919-12.2013 PMC3721838

[16] Lambert JC, Sleegers K, González-Pérez A, Ingelsson M, Beecham GW, et al (2010) The CALHM1 P86L polymorphism is a genetic modifier of age at onset in Alzheimer's disease: a meta-analysis study. J Alzheimers Dis 22: 247–255. 20847397 10.3233/JAD-2010-100933 PMC2964875

[17] Koppel J, Campagne F, Vingtdeux V, Dreses-Werringloer U, Ewers M, et al (2011) CALHM1 P86L polymorphism modulates CSF Aβ levels in cognitively healthy individuals at risk for Alzheimer's disease. Mol Med 17: 974–979. 21629967 10.2119/molmed.2011.00154 PMC3188873

[18] Kauwe JS, Cruchaga C, Bertelsen S, Mayo K, Latu W, et al (2010) Validating predicted biological effects of Alzheimer's disease associated SNPs using CSF biomarker levels. J Alzheimers Dis 21: 833–842. 20634593 10.3233/JAD-2010-091711 PMC3032214

[19] Giedraitis V, Glaser A, Sarajärvi T, Brundin R, Gunnarsson MD, et al (2010) CALHM1 P86L polymorphism does not alter amyloid-beta or tau in cerebrospinal fluid. Neurosci Lett 469: 265–267. 20005921 10.1016/j.neulet.2009.12.011 PMC2860374

[20] Rubio-Moscardo F, Setó-Salvia N, Pera M, Bosch-Morató M, Plata C, et al (2013) Rare variants in calcium homeostasis modulator 1 (CALHM1) found in early onset Alzheimer's disease patients alter calcium homeostasis. PLoS One 8: e74203. 24069280 10.1371/journal.pone.0074203 PMC3775809

[21] Moreno-Ortega AJ, Ruiz-Nuño A, García AG, Cano-Abad MF (2010) Mitochondria sense with different kinetics the calcium entering into HeLa cells through calcium channels CALHM1 and mutated P86L-CALHM1. Biochem Biophys Res Commun 391: 722–726. 19944073 10.1016/j.bbrc.2009.11.127

[22] Gallego-Sandín S, Alonso MT, García-Sancho J (2011) Calcium homoeostasis modulator 1 (CALHM1) reduces the calcium content of the endoplasmic reticulum (ER) and triggers ER stress. Biochem J 437: 469–475. 21574960 10.1042/BJ20110479

[23] Vingtdeux V, Marambaud P (2012) Identification and biology of α-secretase. J Neurochem 120 Suppl 1 34–45. 22121879 10.1111/j.1471-4159.2011.07477.x

[24] Chami L, Checler F (2012) BACE1 is at the crossroad of a toxic vicious cycle involving cellular stress and β-amyloid production in Alzheimer's disease. Mol Neurodegener 7: 52. 23039869 10.1186/1750-1326-7-52 PMC3507664

[25] Stutzmann GE, Mattson MP (2011) Endoplasmic reticulum Ca(2+) handling in excitable cells in health and disease. Pharmacol Rev 63: 700–727. 21737534 10.1124/pr.110.003814 PMC3141879

[26] Cho HJ, Jin SM, Youn HD, Huh K, Mook-Jung I (2008) Disrupted intracellular calcium regulates BACE1 gene expression via nuclear factor of activated T cells 1 (NFAT 1) signaling. Aging Cell 7: 137–147. 18081741 10.1111/j.1474-9726.2007.00360.x

[27] Wen Y, Yu WH, Maloney B, Bailey J, Ma J, et al (2008) Transcriptional regulation of beta-secretase by p25/cdk5 leads to enhanced amyloidogenic processing. Neuron 57: 680–690. 18341989 10.1016/j.neuron.2008.02.024 PMC2329816

[28] Vingtdeux V, Giliberto L, Zhao H, Chandakkar P, Wu Q, et al (2010) AMP-activated protein kinase signaling activation by resveratrol modulates amyloid-beta peptide metabolism. J Biol Chem 285: 9100–9113. 20080969 10.1074/jbc.M109.060061 PMC2838330

[29] Chapuis J, Vingtdeux V, Campagne F, Davies P, Marambaud P (2011) Growth arrest-specific 1 binds to and controls the maturation and processing of the amyloid-beta precursor protein. Hum Mol Genet 20: 2026–2036. 21357679 10.1093/hmg/ddr085 PMC3279048

[30] Exome Variant Server, NHLBI GO Exome Sequencing Project (ESP), Seattle, WA. Available: http://evs.gs.washington.edu/EVS/. Accessed June 2014.

